# Action Recognition Based on Multi-Level Topological Channel Attention of Human Skeleton

**DOI:** 10.3390/s23249738

**Published:** 2023-12-10

**Authors:** Kai Hu, Chaowen Shen, Tianyan Wang, Shuai Shen, Chengxue Cai, Huaming Huang, Min Xia

**Affiliations:** 1 School of Automation, Nanjing University of Information Science and Technology, Nanjing 210044, China; 20211249071@nuist.edu.cn (C.S.); 20211249158@nuist.edu.cn (T.W.); 202312220026@nuist.edu.cn (S.S.); 202183240085@nuist.edu.cn (C.C.); xiamin@nuist.edu.cn (M.X.); 2 CICAEET, Nanjing University of Information Science and Technology, Nanjing 210044, China; 3 Department of Physical Education, Nanjing University of Information Science and Technology, Nanjing 210044, China; 850028@nuist.edu.cn

**Keywords:** skeleton action recognition, temporal modeling, prior knowledge

## Abstract

In action recognition, obtaining skeleton data from human poses is valuable. This process can help eliminate negative effects of environmental noise, including changes in background and lighting conditions. Although GCN can learn unique action features, it fails to fully utilize the prior knowledge of human body structure and the coordination relations between limbs. To address these issues, this paper proposes a Multi-level Topological Channel Attention Network algorithm: Firstly, the Multi-level Topology and Channel Attention Module incorporates prior knowledge of human body structure using a coarse-to-fine approach, effectively extracting action features. Secondly, the Coordination Module utilizes contralateral and ipsilateral coordinated movements in human kinematics. Lastly, the Multi-scale Global Spatio-temporal Attention Module captures spatiotemporal features of different granularities and incorporates a causal convolution block and masked temporal attention to prevent non-causal relationships. This method achieved accuracy rates of 91.9% (Xsub), 96.3% (Xview), 88.5% (Xsub), and 90.3% (Xset) on NTU-RGB+D 60 and NTU-RGB+D 120, respectively.

## 1. Introduction

Human action recognition has been extensively applied in various fields such as video understanding [1], human–computer interaction [2], and virtual reality [3]. Compared to the original RGB video action recognition methods [4], skeleton action recognition approaches, given their explicit inclusion of human joint coordinates, are less affected by lighting or moving backgrounds. Additionally, they exhibit stronger robustness in representing action variations with fewer data. Consequently, the increasing interest in the domain is towards skeleton-based action recognition methods [5,6,7].

Initial skeleton-based action recognition algorithms typically used manual feature extraction methods. They capitalized on geometric transformations to depict the spatial relationships among joints, such as relative positions of the joints [8] and movements of different body parts [9]. However, these techniques often exhibit inadequate generalizability, struggling to capture spatiotemporal features concurrently. In recent years, with the rapid development of deep learning computation [10,11], data-driven approaches have garnered increasing attention, leading to the emergence of Recurrent Neural Networks (RNNs) [12] and Convolutional Neural Networks (CNNs) [13]. RNNs inherently excel at modeling sequential data, making them readily applicable to skeleton-based action recognition. Shahroudy et al. [14] transformed the 3D coordinates of human joints into a time series, with an RNN leveraged for feature extraction. Echoing the approach of [14], a multitude of contemporary methods have adopted RNNs and reported promising outcomes [15,16,17]. Conversely, a CNN can transform skeleton data into pseudo-images to simulate spatiotemporal dynamics.The dual-stream CNN methodology [18] introduces a skeleton transformer module for learning joint representation. However, human skeletal structures cannot be applied directly to methods utilizing RNNs or CNNs as natural graph structures.

Given that skeletal data comprise non-Euclidean structures, the modeling abilities of RNNs [12] and CNNs [13] fall short in capturing inter-joint information. To tackle these issues, Graph Convolutional Networks (GCNs) were introduced to skeleton-based action recognition, yielding excellent results. Yan et al. [5] pioneered the use of GCNs for skeleton data modeling, thereby proposing the Spatio-Temporal Graph Convolutional Network (ST-GCN), and constructed a predefined graph subject to topological constraints. However, the ST-GCN struggles to learn the relationships between skeletal nodes that lack physical connections and do not depend on the data. Hence, 2s-AGCN [19] was proposed, with an adaptive dual-stream graph convolution, allowing new connections beyond natural ones for dynamic graph structure adjustment. The model’s proposed graph topology can be trained end-to-end or independently. Liu et al. [20] proposed a 3D graph convolution, unifying the feature extraction methods of spatiotemporal dimensions for the first time. Zhang et al. [21] enriched node information by introducing joint semantics as an additional feature dimension. Chen et al. [22] proposed a Channel Topology Refinement Graph Convolution Network (CTR-GCN), which captures spatial dependencies between nodes within a channel.

While skeleton action recognition based on GCNs has made some progress in increasing recognition accuracy, the approach still has several drawbacks:

(1). The ST-GCN [5] addressed the challenge of manual graph topology setting by employing a learning-based adjacency matrix method, deploying edge weight multiplication to construct the graph structure. However, the ST-GCN merely forms a graph reflecting natural human connections. It overlooks the links between joints devoid of physical connections. This prevents the addition of new connections to the graph. As its structure is fixed, this might lead to less-than-optimal predictions for samples across diverse action categories. Existing models [19,20,22] fail to make full use of this prior knowledge—the specific movement patterns that the human body follows in daily activities.

(2). As per the human motion coordination theory, encoding processes can track relative movements among body parts while preserving invariance across varying body sizes. Ref. [23] notes that high-order encoding features can be easily incorporated into the existing action recognition framework, complementing joint and skeletal features. However, coordination could be designed to include these higher-order features for understanding motion characteristics, something that is not considered in existing models.

(3). The relationship between the position coordinates of the skeletal nodes is often overlooked, without adequately considering the difference in importance of each skeletal node’s position under different actions. Moreover, it is more appropriate to focus on those frames characterizing representative action features when dealing with a sequence of skeletons.

**Figure 1:**
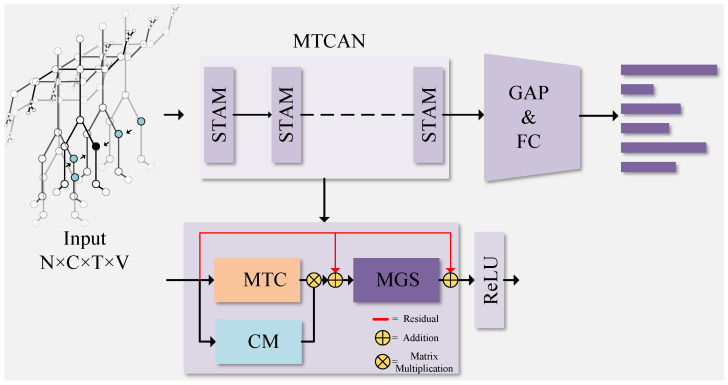
Illustration of the Multi-level Topological Channel Attention Network (MTCAN). The Spatiotemporal Attention Module (STAM) consists of the Multi-level Topological Channel Attention Module (MTC), Coordination Module (CM), and Multi-scale Global Spatiotemporal Attention Module (MGS). The MTCAN is stacked by multiple STAMs (10 layers), and finally the recognition results are obtained through global average pooling and full connection.

This work tackles the aforementioned issues from two angles. Firstly, the spatiotemporal representation learning is bifurcated into spatial and temporal modeling. For spatial modeling, knowledge from daily human activities is used to preprocess and comprehend skeletal data. Studying daily human movements can unveil underlying patterns and laws between diverse behaviors. To achieve this, we present a Multi-level Topological Channel Attention Module (MTC), in combination with a Human Movement Coordination Module (CM). Regarding temporal modeling, we devise a Multi-scale Global Spatiotemporal Attention Module (MGS), leveraging multi-scale temporal convolution. Secondly, robust application of attention mechanisms in spatiotemporal modeling accommodates variations in the significance of spatiotemporal data. The key aspects of these modules can be summarized as follows:

(1). The Multi-level Topological Channel Attention Module (MTC) and the human motion Coordination Module (CM) extract the prior knowledge and coordination features of the human body. These extracted features effectively enhance the base model’s precision across both coarse-grained and fine-grained dimensions.

(2). The Multi-scale Global Spatiotemporal Attention Module (MGS) unifies the causal convolution module and the time attention module with a masking approach, targeting two critical goals. Firstly, this design effectively prevents future information leakage, ensuring that the model can only predict and compute attention using past and present information. Secondly, by introducing the time attention module with a mask, the model can adaptively focus on key feature areas at different time locations, thereby better capturing significant information in the time-series data. This comprehensive attention mechanism endows the model with a stronger expressive ability and better context understanding when processing time-series data. The framework diagram of the proposed Multi-level Topological Channel Attention Network can be viewed in Figure 1.

The structure of this paper is as follows: In the Section 1 we provide a succinct overview of the history of action recognition and the methods previously employed. The Section 2 offers a concise introduction to the concept of skeleton action recognition, as well as knowledge pertinent to spatiotemporal representation learning and attention mechanisms. In the Section 3, detailed descriptions of the three primary modules of the proposed graph convolution skeleton action recognition model are given. The Section 4 presents the effectiveness of the proposed model, validated on three large public datasets, accompanied by ablation and comparative experiments. The Section 5 discusses the proposed model in conjunction with experimental results. The Section 6 summarizes the whole paper and forecasts future research directions.

## 2. Related Work

The subsequent sections delve into the core components of our study’s related work, segmented into the following categories: skeleton-based action recognition, spatiotemporal representation learning, and training/attention measures.

### 2.1. Skeleton-Based Action Recognition

Initial methods of skeleton-based action recognition predominantly relied on manually curated feature descriptors to represent skeleton action sequence characteristics [24,25]. With the emergence of deep learning, action recognition methods based on deep learning have become mainstream, among which the most common ones are recurrent neural networks (RNNs) [14,15,16,26] and convolutional neural networks (CNNs) [18,27]. However, the human skeleton cannot be directly used as a natural graph structure in RNN or CNN methods.

Given that the body’s skeletal structure replicates a natural graph configuration, GCNs have been successfully incorporated into skeleton-based action recognition, producing outstanding results. Yan et al. [5] were the first to apply GCN modeling to skeletal data, proposing the Spatio-Temporal Graph Convolutional Network (ST-GCN) and constructing a predefined graph with topological constraints. However, the ST-GCN finds it challenging to learn the relations between non-physically linked skeletal nodes, and lacks data dependency. Hence, Shi et al. [19] proposed a two-stream adaptive GCN (2S-AGCN), in which the topology of the graph can be uniformly learned through the backpropagation algorithm instead of being set manually. The 2S-AGCN model explicitly combines the second-order information of the skeleton (the length and orientation of the bone) with the first-order information (the coordinates of the joints). Liu et al. [20] introduced 3D graph convolution, marking the first unification of spatiotemporal dimension feature extraction techniques. Zhang et al. [21] enhanced node information by incorporating joint semantics as an additional feature dimension. Chen et al. [22] proposed the Channel Topological Refinement Graph Convolution (CTR-GCN), which was designed to capture the spatial dependencies between nodes within a channel. In particular, the CTR-GC takes the shared topology matrix as a universal prior for channels, and then refines it by inferring channel-specific correlations to obtain the channel topology. Shift-GCN [28], comprising a novel shift graph operation and a lightweight point convolution, provides a flexible receptive field for spatiotemporal graphs. Following this research idea, Song et al. [29] proposed a multi-stream GCN model that fuses input branches including joint positions, motion speeds, and skeletal features in the early stage, and utilizes separable convolutional layers and compound scaling strategies. Redundant trainable parameters are greatly reduced while increasing model capacity. Chi et al. [30] proposed InfoGCN, which includes an information bottleneck objective to learn maximally informative action representations, and an attention-based graph convolution to infer context-dependent skeleton topology. Chi et al. proposed InfoGCN, which includes an information bottleneck objective to learn maximally informative action representations, and an attention-based graph convolution to infer context-dependent skeleton topology.

### 2.2. Spatiotemporal Representation Learning

The paramount task in skeleton action recognition is to extract various behavioral feature information from the skeleton data. In this process, spatial information is extracted through spatial graph convolution, and temporal information is captured through the regular convolution of preceding and following frames. By superimposing the two, spatiotemporal representation learning is achieved. In spatial representation learning, ST-GCN, 2s-AGCN, and others introduce different matrices to encode topological information. Both the method proposed by Cheng et al. [31] and the GCR-GC proposed by Chen et al. [22] set separate parameterized topologies for channel groups, capturing the specific correlations between each channel. Liu et al. [32] proposed the MS-G3D graph convolution model for disentangling and unifying graph convolution, serving as a means for direct information transmission across the spatiotemporal domain. In the temporal domain, ST-GCN, 2s-AGCN, and others employ temporal convolution modules to extract temporal dimension information. The methodologies diversify beginning with TCN [33], MG-G3D, and AML-GCN [34], with a widespread adoption of multi-scale temporal convolution. In NAS-GCN [35], a Gaussian function is introduced to compute node correlations.

### 2.3. Training/Attention Measure

The attention mechanism is employed to allot varying degrees of focus to information across different dimensions, time, and space, leveraging more feature information to play a pivotal role in the recognition process and mitigating the impact of information with limited degrees of differentiation. Therefore, an increasing number of attention mechanisms are being implemented in skeleton action recognition to enhance recognition performance. Liu et al. [36] proposed two distinct attention aggregation strategies in their study, viewing facial key points as nodes and dynamically updating each node’s visual attributes by considering the inter-nodal pose and position relationships. A multi-level attention mechanism was introduced to help the model focus on information components during the learning of representations. Liu et al. [37] proposed GCA-LSTM, a method with a recurrent attention mechanism for global context awareness, designed for the handling of skeletal sequences. This mechanism dynamically updates the attention weights at each timestep to better selectively focus on the information joints within frame sequences.

In pursuit of effective model architectures, this study delves into the attention mechanism widely used in the field of natural language processing. Hu et al. [38] first put forward the SE-net model that compresses features in the spatial dimension and uses Multilayer Perceptron (MLP) [39] to explicitly model channel correlations. Additionally, Woo et al. [40] proposed the CBAM model, which employs an attention-based feature refinement technique to consider both the channel and spatial dimensions. In contrast to the aforementioned methods, this paper, leveraging the specificity of skeletal data, designs a level topological channel attention module that combines human skeleton prior knowledge with channel attention, from a coarse to fine level, to delineate the varying significance of different body parts.

In order to achieve competitive recognition results, multi-feature fusion methods are widely used in deep learning tasks [41,42,43]. Simonyan et al. [44] proposed a dual-stream network, composed of two structurally identical branches: a temporal stream and a spatial stream. Each branch independently trains for action recognition before the classified scores of the two branches are finally fused to obtain the final prediction result. Wang et al. [45] proposed a tri-stream convolutional network based on the dual-stream structure, where the temporal stream is subdivided into local and global temporal streams. In skeletal action recognition, Shi et al. [19] used joint features as first-order information and skeletons comprising direction and length as second-order information, constructing a dual-stream structure for late fusion. During the training, Le et al. [46] further deployed a quad-stream fusion, achieving excellent recognition accuracy. Hence, this paper also adopts the strategy of training with a four-stream structure suitable for late fusion.

## 3. Methodology

In this section, we first review the sequential representation method of skeleton action recognition and spatiotemporal graph convolution operators. Following this, we provide a detailed description of the multi-level topological channel module based on attention and the multi-scale global spatiotemporal module.

### 3.1. Preliminaries

#### 3.1.1. Skeleton Sequence Representation

The original skeleton sequence consists of a series of coordinate data, which can be represented by the 3D joints of the human body in each video frame. Since the topological structure of the human skeleton is a natural graph, it allows skeleton-based human actions to be represented as spatiotemporal graphs. The ST-GCN [5] is the earliest graph neural network that utilized spatiotemporal graphs for modeling skeletal points in time and space dimensions. Specifically, an undirected graph G=(V,E) is constructed on the skeleton sequence *X*, which comprises *N* skeletal nodes and a time length of *T*. The set of nodes can be expressed as:

(1) $$V=\{v_{ti}|t=1,2,3,\ldots \ldots T;i=1,2,3,\ldots \ldots N\}$$

Here, *V* denotes the set of nodes, $v_{ti}$ represents the ith skeleton point in the tth frame, and *V* includes all nodes in the skeleton sequence *X*. The skeleton edge set *E* consists of skeleton edge set $E_{S}=\left\{v_{ti}v_{tj}|\left(i,j\right)\in H\right\}$, which connects various skeletal points within the same frame and skeleton edge set $E_{F}=\left\{v_{ti}v_{(t+1)i}\right\}$ that links the same skeletal points between successive frames, where H represents the naturally connected human skeletal joints.

According to the defined graph G, the spatial graph convolution operator, in terms of spatial dimensions, is represented as:

(2) $$f_{out}=\Lambda^{\left(-1/2\right)}\left(A+I\right)\Lambda^{\left(-1/2\right)}f_{in}⊗W_{S}$$

Here, $f_{in}$ denotes the input skeletal sequence of dimensions $C_{in}\times T\times N$; $f_{out}$ signifies the output skeletal sequence of dimensions $C_{out}\times T\times N$; ⊗ is used to represent the convolution operation, while $W_{S}$ signifies the spatial convolution kernel with dimensions $C_{in}\times C_{out}\times 1\times 1$; *A* also refers to the adjacency matrix with dimensions of *N* × *N*.

(3) $$A\left[i,j\right]=\left\{\begin{array}{c} 0,v_{ti}v_{tj}\notin E_{S}\left(\tau \right)\\ 1,v_{ti}v_{tj}\in E_{S}\left(\tau \right) \end{array}\right.$$

The time graph convolution operator within the temporal dimension shares similarities with the classic 2D convolution operation. This is due to each vertex $v_{i}$, whose corresponding joint vertices on two adjacent continuous frames remain consistent, meaning it possesses two neighboring nodes on the timeline. The time graph convolution operator is represented as:

(4) $$f_{out}=\Lambda^{\left(-1/2\right)}\left(A+I\right)\Lambda^{\left(-1/2\right)}f_{in}⊗W_{F}$$

Here, $W_{F}$ denotes the time graph convolution kernel with dimensions $C_{in}\times C_{out}\times T\times 1$, which represents the trainable parameters of the time graph convolution kernel.

#### 3.1.2. Dataset

NTU-RGB+D 60 Dateset [14]: The NTU-RGB+D 60 Dataset is a publicly available large dataset tailored for action recognition based on 3D skeletons. It comprises 56,578 action sequences, spanning across 60 categories of everyday interactions, which include individual actions, interactions with objects, and between people. The model is evaluated using two benchmarks: cross-subject (xsub) and cross-view (xview). For the cross-subject, 3D skeleton sequences from 20 specific actors’ IDs are used for training, with the remaining samples used for testing. For cross-view, it utilizes the skeleton data from three cameras, where cameras 2 and 3 are used for training, and camera 1 for testing.

NTU-RGB+D 120 Dateset [47]: The NTU-RGB+D 120 Dataset is an extension of the NTU-RGB+D 60 Dataset, encompassing 113,945 skeletal sequences, which cover a more diverse range of everyday activities, totalling up to 120 categories. Specifically, this dataset encompasses skeletal sequences from 106 performers of varying ages, is set in 32 different scenes, and involves 155 camera views. The dataset has two conventional evaluation criteria: cross-subject (xsub), whereby the skeletal data from 53 specific performer IDs are used for training, with the remaining samples for testing, and cross-setup (xset), where even IDs are designated for training and odd IDs for testing.

NW-UCLA [48]: The NW-UCLA dataset includes 1497 videos of 10 different types of actions, captured simultaneously from three cameras. In this paper, the data from the first two cameras are used for training, while the remaining data are employed for testing, following the methodology outlined in [48].

#### 3.1.3. Experimental Settings

All experiments in this paper were conducted using the Pytorch deep learning framework on an RTX3080 12 g graphics card, with Python version 3.9 and Pytorch version 9.1. All models utilized Stochastic Gradient Descent (SGD), with a momentum of 0.9, weight decay of 0.0004, batch size of 64, and an initial learning rate of 0.1. The Cross Entropy loss function was employed for a total of 65 epochs. The learning rate was divided by 10 at the 35th and 55th epochs. A warm-up strategy was employed during the first 5 epochs to stabilize the training process. For NTU-RGB+D 60 and NTU-RGB+D 120, the preprocessing method from [22] was applied to adjust each skeleton sequence to 64 frames. For the NW-UCLA dataset, the batch size was set to 16 and the preprocessing method from [28] was utilized. Additionally, four data modalities were set up for training: joint, bone, joint-motion, and bone-motion. The performance of the four modalities was then integrated to obtain the final accuracy. To enhance the reliability of the experimental results, the ablation and comparison experiments described in Section 4.2 and Section 4.4 of this paper were repeated 10 times each during the training process. The final result for all experiments in this paper was computed as the average of the outcomes from the 10 repetitions.

The experimental evaluation metric is defined as the probability of correctly identifying all actions, that is, accuracy. Since all the classes are equally essential, it is widely employed. It can be defined by the following formula:

(5) $$Accuracy=\frac{TP+TN}{TP+TN+FP+FN}$$

TP (True Positive) is the number of true positives, that is, the number of samples correctly identified as positive. TN (True Negative) is the number of true negatives, that is, the number of samples correctly identified as negative. FP (False Positive) is the number of false positives, that is, the number of samples incorrectly identified as positive. FN (False Negative) is the number of false negatives, that is, the number of samples incorrectly identified as negative. This formula takes into account all possible classification results and calculates the accuracy across all test samples.

### 3.2. Multi-Level Topological Channel Attention Network (MTC)

This module models the channel relationships of the input skeletal feature X and the coordination of human limbs in kinematics. This paper divides the prior knowledge of human motion into two categories:

(1). From a detailed perspective, human motion is carried out on a limb-by-limb basis. This section depicts the relationship between individual limb movements and overall body motion.

(2). From a coordination standpoint, human motion involves inter-limb movements. This section articulates the relationship between the movements of different limbs.

#### 3.2.1. The Multi-Level Topological Channel Attention Module (MTC)

Following the laws of human motion, this paper categorizes the human skeletal structure into two hierarchical levels. The first level divides the body into two segments: the upper body, consisting of everything above the last lumbar vertebra, and the lower body, consisting of everything below it. The second level consists of four parts: the left arm, right arm, left leg, and right leg. Channel attention initially calculates the attention on the dimension of the first-level topological structure, rendering a coarse-grained representation in the feature map. Subsequently, based on the coarse-grained information, it computes the attention on the second-level topological structure’s channel dimension, producing a finer-grained representation in the feature map.

**Figure 2:**
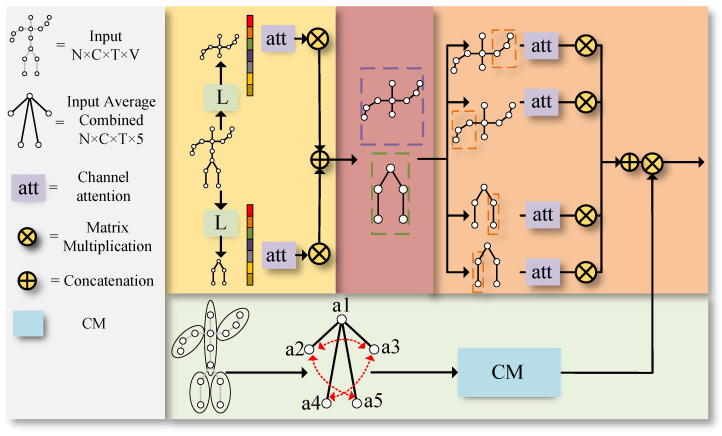
The framework of the Multi-level Topological Channel Attention Module (MTC).

Initially, as shown in Figure 2, we used an action recognition dataset based on human bone structure as input. This dataset is carefully processed and consists of human skeleton data. The dimensions of the input original data are *N* × *C* × *T* × *V*, where *N* represents the batch size, *C* represents the number of channels, *T* represents the timing length, and *V* represents the number of bone points. The figure shows the channel attention module “att” used in Multi-level Topological Channel Attention, forming a multi-level topology. Additionally, the lower part of the figure shows the Coordination Module. The output of the model is jointly weighted by the Multi-level Topological Channel Attention Module and the Coordination Module.

**Figure 3:**
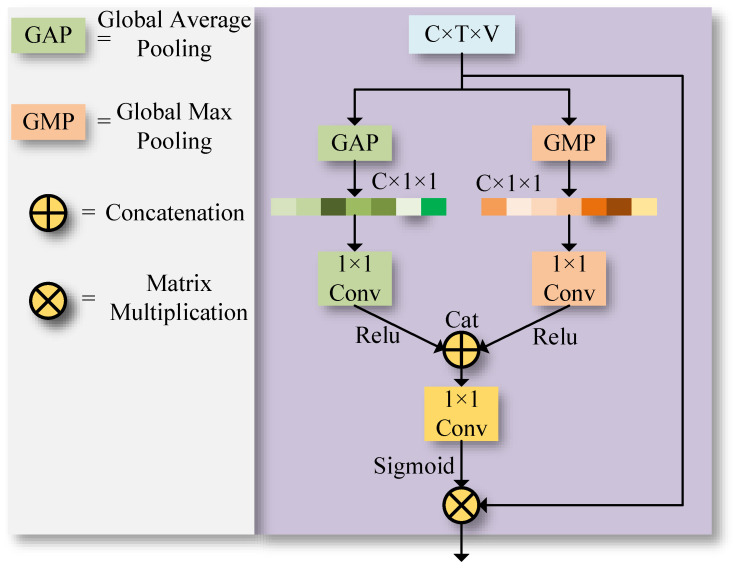
Illustration of the channel attention model.

As depicted in Figure 3, both global average pooling and global max pooling layers are employed for extracting advanced topological features. Various types of global pooling layers can extract a wealth of topological features. The skeletal data $X_{input}$, input in the shape of C×T×V, where *C* denotes the number of channels, *T* the sequence length of the skeleton, and *V* the number of skeleton joints, will yield channel features of shape C×1×1 after processing through two global pooling layers. The channel features are then dimensionality-reduced by a convolutional layer with a kernel of 1×1. The process can be represented as:

(6) $$g_{Gap}=\frac{1}{N\times T\times V}\sum_{i=1}^{N}\sum_{j=1}^{T}\sum_{k=1}^{V}X_{input}\left(i,j,k\right)$$

(7) $$g_{Gmp}=\max\left\{X\left(i,j,k\right)\right\}$$

where i=1,2,……,N; j=1,2,……,T; k=1,2,……,V.

(8) $$X_{Gap}=Cov\left(g_{Gap}\left(x_{i}\right)\right)$$

(9) $$X_{Gmp}=Cov\left(g_{Gmp}\left(x_{i}\right)\right)$$

Here, $g_{Gap}$ and $g_{Gmp}$ represent average pooling and global max pooling, respectively, while *i*, *j*, and *k* denote positions in the *N*, *T*, and *V* dimensions. Conv stands for a convolution layer with a kernel size of 1. $X_{Gap}$ and $X_{Gmp}$ represent the extracted global average pooling and global max pooling features, respectively. The outputs from these two types of global pooling are concatenated and fed into a convolution layer with a kernel size of 1. This convolution layer serves as a selector, adept at adaptively focusing on the features represented by the two types of global pooling. Finally, the features are reweighted using a sigmoid activation function. This process can be illustrated as follows:

(10) $$U_{channel}=X_{input}\cdot Sigmoid\left(Conv\left(Cat\left(X_{Gap},X_{Gmp}\right)\right)\right)$$

In this context, Cat denotes the concatenation operation, Sigmoid refers to the activation function, and Conv stands for a convolution layer with a kernel size of 1. $X_{input}$ represents the input, and the final outcome, $U_{channel}$, refers to the features weighted by channel attention

The Multi-level Topological Channel Attention Module first uses a feature linear transformation layer to convert the input features $X_{input}\left(N,C,T,V\right)$ into U(N,C,T,V), thereby extracting high-level representations:

(11) $$U=L\left(X_{input}\right)=X_{input}W$$

Afterwards, using a predefined first-level topology structure, U(N,C,T,V) is transformed into $U1(N,C,T,V_{upper})$ and $U2(N,C,T,V_{dower})$. Subsequently, *U*1 and *U*2 are separately fed into the channel attention modules, yielding two channel feature descriptors, $U1_{channel}$ and $U2_{channel}$. Finally, the Cat operation is employed to concatenate these two descriptors, forming the channel feature descriptor $U_{Level1}$ with a first-level topology structure:

(12) $$U_{Level1}=U1_{channel}+U2_{channel}$$

Following the aforementioned process, we obtain a feature map of coarse granularity. Subsequently, treating $U_{Level1}$ as the input, we divide it into four parts according to a predefined partition, $U_{1}$, $U_{2}$, $U_{3}$, and $U_{4}$, corresponding respectively to left hand, right hand, left leg, and right leg. We then repeat the mentioned formula, to calculate the fine-grained attention for each part within the channels. This results in obtaining the mixed channel feature $U_{i},i\in \left[1,4\right]$. This defines a secondary topology as follows:

(13) $$U_{Level2}=U_{1}+U_{2}+U_{3}+U_{4}$$

#### 3.2.2. Coordination Module (CM)

**Figure 4:**
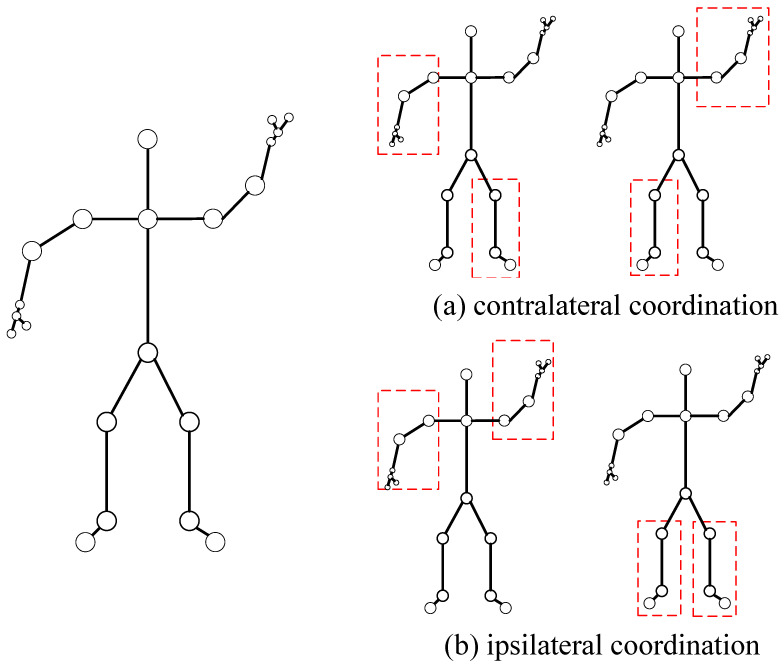
Movement coordination diagram where (**a**) is contralateral coordination and (**b**) is ipsilateral coordination. Human body parts framed in red to show two different types of movement.

Even though this paper achieved a high accuracy in ablation experiments using first-order and second-order features of skeletons (namely, $U_{Level1}$ and $U_{Level2}$, derived from Formulas (11) and (12) in Section 3.2.1, correspond to the first-order and second-order information, respectively), the similar motion trajectories of type actions still lead to misjudgments. Hence, it becomes necessary to obtain higher-order features to support the lower ones. A person always maintains balance during motion, which requires dynamic coordination between the limbs. From a coordination perspective, human motion involves inter-limb movements, i.e., the relationship between limb movements. As shown in Figure 4, in the realm of human kinetics, motion or types of motion are typically classified into contralateral coordination, as shown in (a), usually contralateral movements of the left hand and right foot (such as running and walking) and ipsilateral coordination, as shown in (b), through ipsilateral movements of the left and right hands (such as swimming and Tai Chi). Both are used to describe the coordinated movements between limbs. Therefore, this study aims to construct a coarse-grained ratio graph to extract the coordination characteristics between limbs and apply weights to the original skeleton by generating a coordination matrix.

**Figure 5:**
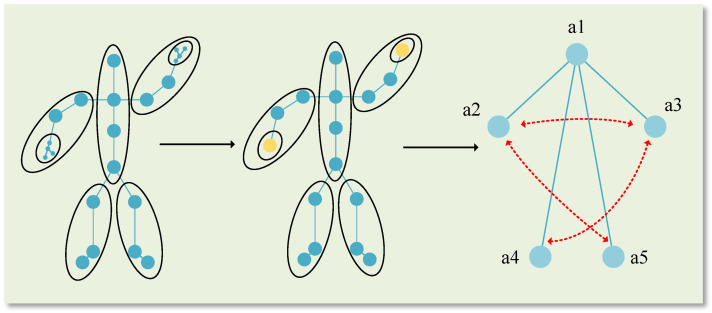
Schematic diagram of a coarse-grained scale map. The first is the original human skeleton diagram, then the coordinate averaging method is used to divide the finger parts first, and finally the human skeleton is divided into five parts.

Next, using the given skeleton sequence X, we construct a coarse-grained proportion map. The human skeleton is divided into five parts: the central torso, the left arm, the right arm, the left leg, and the right leg. Each of these parts is processed separately. Typically, the most common method is to calculate the centroid of each region to represent its approximate location. However, this method can be flawed for non-convex shapes, such as a bent arm. A protrusion at the elbow, or any indentations, might lead to offsetting the centroid coordinates, thereby failing to accurately depict the features of that part. Consequently, this study adopts the mean coordinate method, which is applicable to various non-convex shapes present in skeleton data, thereby preserving more detailed information. The positions of the skeleton points included in each part are processed by calculating the mean coordinates, which are then merged into new skeleton points. This is referred to as the coarse-grained proportion map. Figure 5 illustrates the graph structure constructed corresponding to the coarse-grained proportion map.

In biomechanics, movements or types of movements are typically classified as contralateral or ipsilateral coordination. Therefore, in the coordination module, correlation coefficients for the left and right arms (a2, a3), the left arm and right leg (a2, a5), and the right arm and left leg (a3, a4) in the coarse-grained proportion map are calculated separately (in the analysis of movement types in the dataset, instances of ipsilateral coordination involving both legs are sparse, which is to say, if the hands are coordinated, so are the legs). First, calculate the Euclidean spatial distances between a2 and a3, a2 and a5, and a3 and a4 to obtain the distance parameters *d*1, *d*2, and *d*3. These are subsequently processed using exponential weighting and normalization. Figure 5 below represents a schematic of the coarse-grained proportion map:

(14) $$w_{1}=\frac{\exp(-d1)}{\exp(-d1)+\exp(-d2)+\exp(-d3)}$$

(15) $$w_{1}=\frac{\exp(-d2)}{\exp(-d1)+\exp(-d2)+\exp(-d3)}$$

(16) $$w_{1}=\frac{\exp(-d3)}{\exp(-d1)+\exp(-d2)+\exp(-d3)}$$

In which *w*_1_, *w*_2_, and *w*_3_ represent the coordination correlation coefficients. A weighted calculation is performed on the skeleton as follows: $X_{cm}=\sum_{i=1}^{3}d_{i}\cdot X$. $X_{cm}$ denotes the weighted skeleton sequence.

### 3.3. Multi-Scale Global Spatiotemporal Attention Module (MGS)

**Figure 6:**
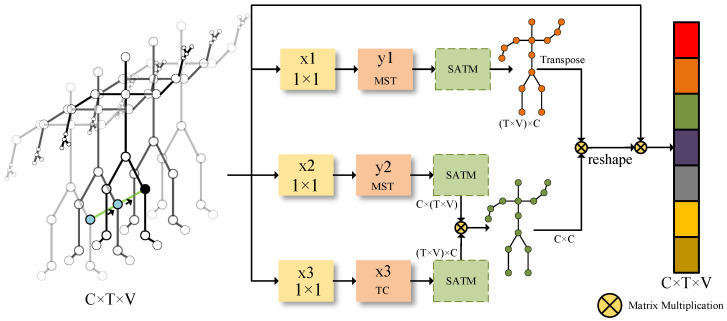
The framework of the Multi-scale Global Spatiotemporal Attention Module (MGS).

Since the spatial graph convolutional layer only aggregates information in space, it cannot effectively interact with information in the time window. Thus, it is necessary to model the time dimension features of the skeleton sequence. This paper designs a Multi-scale Global Spatiotemporal Attention Module, which employs multi-scale time graph convolutional layers for multi-branch expansion, captures spatiotemporal patterns of different feature granularities, and calculates the correlation between the current feature’s position and other spatiotemporal positions to capture the global dependencies among spatiotemporal features. The network model is illustrated in Figure 6:

The Multi-scale Global Spatiotemporal Attention Module (MGS) first takes in the *X* skeleton feature with a shape of C×T×V, passing it through three convolutional layers with a kernel size of 1, yielding x1, x2, x3 $\in R^{C\times T\times V}$, where *C* denotes channels, *T* stands for skeleton temporal scale, and *V* represents the number of skeleton joints. Inputs x1 and x2 are entered into the Multi-scale Time Convolutional Layer that contains depth-wise causal convolution blocks, resulting in *y*1 and *y*2. Next, *y*1, *y*2, and x3 are subjected to matrix transformations, resulting in $y1\in R^{K\times C}$, K=T×V; $y2\in R^{C\times K}$, K=T×V; and $x3\in R^{K\times C}$, K=T×V. The relationship between the current spatiotemporal features and others is calculated by performing matrix multiplication on *y*2 and x3, followed by a Softmax operation to obtain the global spatiotemporal attention weight coefficient $S\in R^{C\times C}$. After obtaining the global attention weight coefficient, $SA\in R^{K\times C}$ is obtained by element-wise multiplication with *y*1, resulting in an attention-inclusive feature map. Finally, the features are passed on to the output of the module by way of residual connection with the input features.

**Figure 7:**
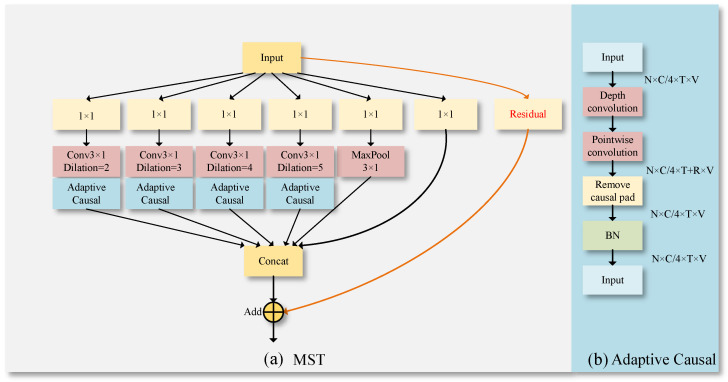
Illustration of the MST model. (**a**) is the Multi-scale Temporal Graph Convolution Layer improved in this article, and (**b**) is the adaptive causal convolution used in MST.

**Figure 8:**
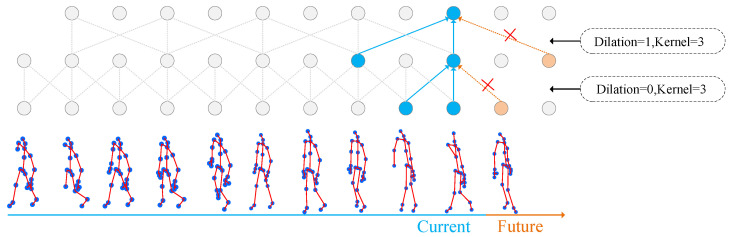
Schematic diagram of timing information leakage.

The MST module is shown in Figure 7; this paper introduces improvements to the classic Multi-scale Temporal Graph Convolution Layer (MSTGCL). Behind these improvements, we became aware of a specific scenario where using the MSTGCL might lead to leakage of future information due to different dilation rates, as depicted in Figure 8. The leakage of future information is an issue that must be avoided when dealing with temporal data. “Information leakage into the future” simply refers to a scenario where the model acquires data during temporal convolution that it should only be able to access in the future. Dilated convolution plays a crucial role in the MSTGCL. It allows for the calculation of convolution at different dilation rates, thus capturing patterns in the input data at varied scales. Thus, as shown in Figure 7b, we implemented a deep causal convolution module within the dilated convolution. It ensures that data convolution is performed under the premise of causal relationships.

Specifically, when performing convolution operations on the temporal dimension T, disregarding the causal relation on the time axis—that is, if the convolution kernel can access data beyond the current timestep—can lead to leakage of future information. To mitigate this issue, we adopt a unique padding method ensuring that the convolution kernel only has access to current and past data, eliminating potential access to future temporal information and maintaining causality. As per Figure 7b, the skeleton features of shape C/4×T×V are input into the depth convolution. Within the depth convolution, each channel convolves only with itself, reducing parameter and computational demands. Next, the inputs are directed into pointwise convolution, where the output channels are merged within the pointwise convolution. Thereafter, a two-dimensional convolution layer is defined in “Remove causal pad”, setting the padding parameters, dilation for specifying the convolution kernel dilation factor, and stride for determining the convolution step length. Input data of varying scales are first convolved through the convolution layer. The remove operation is then used to eliminate the outputs queued behind by R timestep lengths, where the value of remove equates to R=pad/stride. Lastly, the output is returned after normalization using Batch Normalization (BN).

The MST module adopts a bottleneck design, thereby reducing the parameter count to a certain extent. As per Figure 7a, six branches were designed. Each utilizes a temporal graph convolution kernel with a dimension of C×C/6×1×1 to reduce the channel dimension of the skeleton sequence to C/6 to minimize computational complexity. Four branches respectively employ temporal graph convolution kernels with dimensions set as $C_{origin}\times C_{out}\times 3\times 1$ and dilation rates of 2, 3, 4, and 5 to extract multi-scale temporal features from the skeleton sequence. Moreover, to expand the receptive field of the model, an additional temporal graph convolution and max pooling branch with a dimension of C×C/6×1×1 has been included. Finally, the Concat operation is used to restore the channel dimension to C. A type of residual connection is introduced to optimize gradient propagation, resulting in the output of the multi-scale temporal graph convolution layer.

In the Multi-scale Global Spatiotemporal Attention Module (MGS), Self-Attention Temporal Module (SATM) is introduced, which includes self-attention with a time mask and time convolution network. In this context, this paper opts to employ masked time attention. One reason for this is to enable the model to adaptively focus on key feature areas of different time positions as needed. This allows for the extraction of features in the most effective manner, and also enhances the model’s performance with limited parameters. On the other hand, the masked time attention can obscure information for a specific time moment, thereby preventing data leakage.

**Figure 9:**
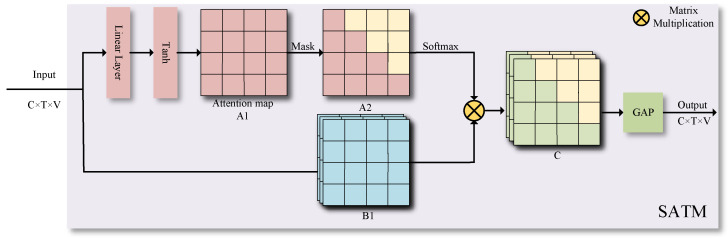
Illustration of the Self-Attention Temporal Module (SATM).

Specifically, the Self-Attention Temporal Module (SATM) takes a skeleton sequence with dimensions C×T×V as input. Initially, the input goes through a linear transformation layer and is then plugged into the Tanh nonlinear mapping function, obtaining the attention distribution at different time points with dimensions T×1×V. Next, this attention distribution is replicated T times to yield the attention distribution matrix A1 with dimensions T×T×V, as depicted in Figure 9.

Concurrently, the input features undergo the same processing to result in the feature matrix B1 with dimensions C×T×T×V. Next, a masking operation is performed on the upper right corner of attention matrix A1 by filling it with negative infinity, thus creating the masked attention distribution matrix A2. Thus, after passing through the Softmax function, the weight coefficients corresponding to the masked part will become zero, thereby preventing the model from focusing on future information. Subsequently, the masked attention distribution matrix A2 and feature matrix B1 are matrix-multiplied to generate the time-varied feature matrix C with dimensions C×T×T×V. Finally, a global average pooling operation is applied on feature matrix C. By averaging the weighted feature at each moment, an output of dimensions C×T×V is obtained. The weighted feature at each moment is obtained through the time-attentive weighting of past features. With this processing procedure, the SATM module can adaptively focus on the key feature regions at different time positions and extract more effective feature representations using limited parameters.

## 4. Experimental Results

### 4.1. Module Ablation Study

To verify the effectiveness of the methods presented in this paper, ablation experiments were carried out on the NTU-RGB+D 60 dataset for the Multi-level Topological Channel Attention Module (MTC), Coordination Module (CM), and Multi-scale Global Spatiotemporal Attention Module (MGS). The main evaluation criterion adopted in this paper is the Recognition Accuracy Rate.

Multi-level Topological Channel Attention Module (MTC): To verify the effectiveness of this attention model on skeleton data, we removed it from the network architecture. As seen from Table 1, the MTC achieved a top-1 accuracy improvement of 0.7% on Xsub, and a top-1 accuracy improvement of 0.5% on Xview. Due to the multi-level topological structure adopted in the MTC, which combines coarse-grained and fine-grained information, it assists in capturing more delicate human physical characteristics.

Coordination Module (CM): To validate the effectiveness of the coordination module, this study incorporates features extracted by it into the model and conducts joint training with bone and joint. Our baseline represents the precision without using the coordination module. From Table 1, we observe a performance improvement of 0.3% in top-1 precision on Xsub, and 0.2% on Xview, achieved through our coordination module. Additionally, to investigate the fine-tuned enhancement of the coordination module for specific actions within NTU-RGB+D 60, we extract 10 actions and discuss their improvements in both bone and joint streams, respectively. As shown in Table 2, the cascading of the coordination module and skeletal features has resulted in significant enhancements for the bone stream. For the joint stream, although the precision in some parts is lower, the coordination features have nevertheless contributed to a noteworthy improvement.

**Figure 10:**
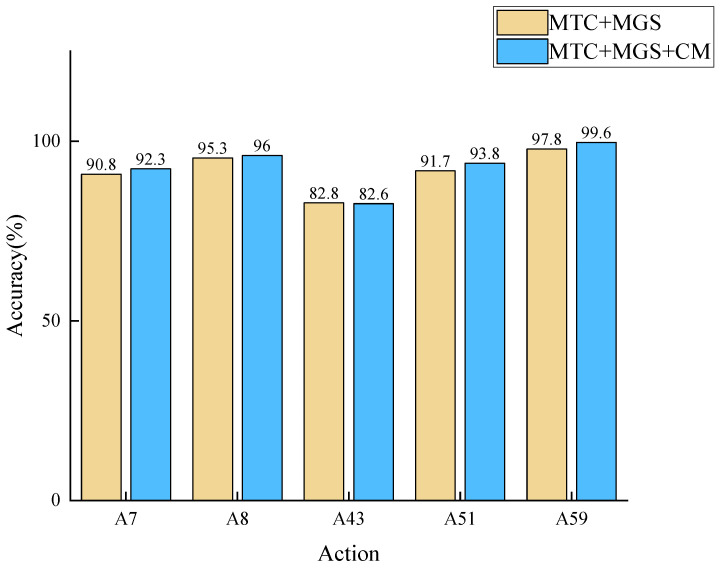
Five different types of actions were tested on the joint flow of NTU-RGB+D 60.

Furthermore, this paper individually verifies the benefits that the CM module brings for specific actions. As illustrated in Figure 10, five different types of actions are tested on the joint stream of NTU-RGB+D 60. Except for A43, all other actions have shown improvement (A7.throw, A8.sitting down, A43.falling, A51.kicking other person, A59.walking towards each other). The accuracy decline for A43 might be due to it not being a contralateral or ipsilateral coordination movement. In conclusion, the coordination module is capable of effectively extracting human motion coordination features, which can be more evidently demonstrated in some specific actions.

Multi-scale Global Spatiotemporal Attention Module (MGS): Multi-scale modeling is a common issue in the field of computer vision. MGS proposed a Multi-scale Global Spatiotemporal Attention Module, equipped with a multi-scale causal convolution module and a time-attention module with a mask. As seen from Table 1, MGS significantly improved the feature representation of the module. This shows that MGS can effectively simulate the spatial location differences between various actions. Ultimately, this paper finds that combining MTC, CM, and MGS can achieve the best recognition performance. This paper will visualize the focus of Multi-scale Global Spatiotemporal Attention Module (MGS) on different actions in Section 4.4 to verify their effectiveness.

### 4.2. Ablation Experiments within a Single Model

This section conducts ablation experiments for the CM and MGS modules mentioned in Section 3 separately. Here, we first compared the methods of constructing coarse-grained ratio maps in the CM module, choosing three methods for comparison: sampling, finding the centroid, and averaging. As shown in the Table 3, the averaging method is slightly superior to the centroid-finding method, exceeding it by 0.4% and 0.1% on NTURGB60 and NW-UCLA, respectively. However, the sampling method did not yield the desired results. Since different coordinate points represent different features and properties, the sampling method may not be able to fully capture all the information. The centroid-finding method could lead to a shift in the center of mass due to the non-convex shape of the limb. The averaging method employed in this paper can achieve the best results.

Next, the MST and SATM modules within the MGS module were subjected to ablation studies. The experimental results are shown in Table 4. The table suggests an improvement of 1.1% due to the MST module and 0.2% due to the SATM module in the NTU-RGB+D 60 dataset, indicating that the MST module contributes more than the SATM. Conversely, in the NW-UCLA dataset, the impact of the SATM is greater than that of the MST. These results not only confirm the significant role of the MST and SATM modules in our model, but also highlight the variance in their effect across different datasets. When tackling diverse recognition tasks, the effectiveness of MST or SATM modules can be selectively enhanced based on the dataset. This provides a flexible optimization strategy. By integrating modules with different characteristics, the model proposed in this paper can effectively adapt to various datasets and task environments.

### 4.3. Multi-Stream Framework Verification

**Figure 11:**
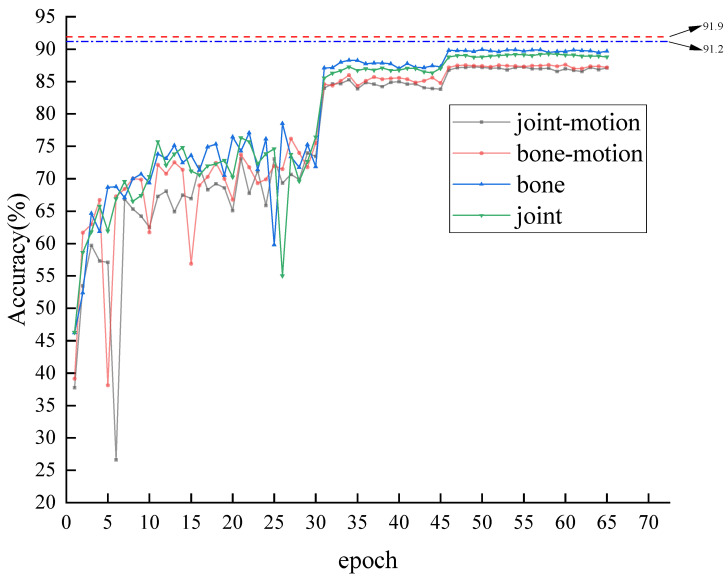
The training process of joint flow, bone flow, joint-motion flow, and bone-motion flow, as well as the fusion results of dual-stream and four-stream.

During the training of the model, a multi-stream strategy was employed, utilizing joint, bone, joint-motion, and bone-motion to represent joint morphology, bone morphology, joint movement, and bone movement, respectively. These four data schemes were examined. As shown in Figure 11, an analysis of various data models was conducted on the xsub protocol of NTU-RGB+D 60. The four training curves sequentially represent joint, bone, joint-motion, and bone-motion. Moreover, the red dotted line and the blue dash-dot line at the top symbolize the four-stream fusion and the dual-stream fusion of “joint” and “bone”, respectively. It is observable that the performance of bone stream slightly exceeds that of joint stream and the bone-motion stream performs marginally better than the joint-motion stream. The multi-stream fusion invariably outperforms the four individual streams, with the accuracy rate of four-stream fusion being 91.9%, surpassing the duo-stream fusion rate of 91.2%. This indicates that each single stream exhibits superior performance in recognizing different movements. Multiple streams, complementing each other, will bring further improvement when combined.

### 4.4. Visualization

**Figure 12:**
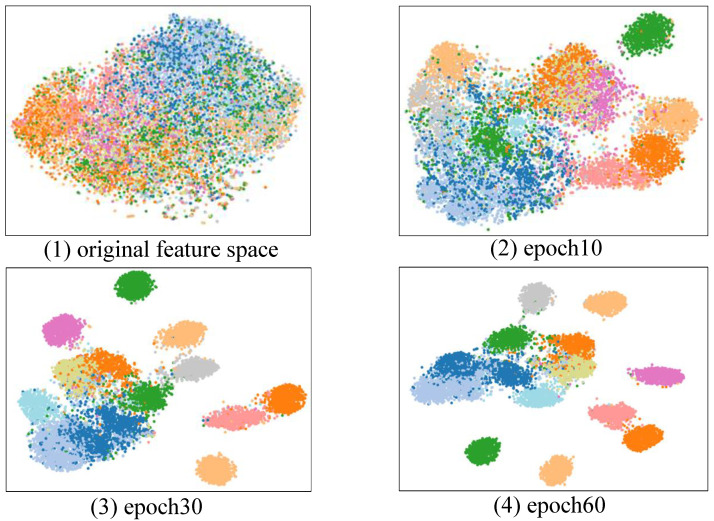
T-SNE dimensionality reduction visualization. Dots of different colors represent different action types. The better the clustering effect of dots of the same color, the better the classification effect.

To thoroughly evaluate the effectiveness of our proposed model, this section begins with a deep quantitative analysis including Principal Component Analysis (PCA), focusing on the representational characteristics of various actions. For a more intuitive presentation of these results, we randomly selected fifteen types of actions from NTU-RGB+D 60 for feature distribution visualization. As demonstrated in Figure 12, the model extracts both the original feature distribution as well as feature distributions from the 10th, 30th, and 50th training rounds. Each point represents an action sample, and each cluster represents a type of action, demonstrating the degree of feature refinement by the model in different training rounds. It can be seen that with the increasing number of training rounds, these clusters (i.e., the features of each type of action) gradually become more compact and dispersed, indicating that the model can effectively learn and extract features of actions, creating clearer boundaries in the feature space for different actions. Therefore, during the training process, the feature space gradually presents a more precise and non-overlapping feature distribution, becoming increasingly distinctive. This suggests that our model’s capability in feature extraction and action segmentation is steadily improving.

**Figure 13:**
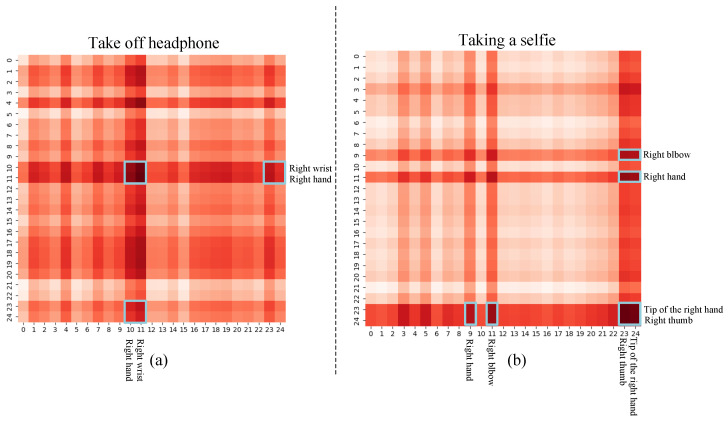
Visualization of the connection strength of two action examples. (**a**) Indicates the action “Take off headphone“. The parts enclosed by blue boxes are represented as Right wrist and Right hand to indicate that this action has a more important role in this part. (**b**) Indicates the action “Taking a selfie”. The parts enclosed by blue boxes are represented as Right bebow, Right hand, Tip of the right hand and Right thumb to indicate that the action has a more important role in this part. It is observed that our model can adaptively focus on joints that are highly relevant to actions without relying on prior physics knowledge.

Additionally, this paper explores the influence of the prior knowledge module and the coordination module on inherent topological behaviors in humans, thereby aiding scholars in better understanding how the model distinguishes between human behavioral patterns. Here, the paper presents visualizations of adjacency matrix illustrations for two actions to analyze their internal topological structures. Figure 13 displays the connectivity learned by the model. The x and y coordinates represent the 25 skeletal points of human connection in the NTU-RGB+D 60 dataset. The depth of color in the heatmap indicates the strength of skeletal connection. The action “Take off headphone” typically requires a connection between the hand and wrist. Figure 13a displays that the adaptive graph learned by the model primarily correlates between right hand and right wrist, with these joints all within right hand. This association reflects that the performer is accustomed to using the right hand to remove headphones. The action “Taking a selfie” commonly requires holding a phone with one hand, bending the elbow, and using fingers for operation. Figure 13b shows that the main connections learned by the model are located at the right elbow, right hand, right fingertip, and right thumb. The connection of these four areas indicates that the performer uses the right hand for the action of taking a selfie. These results suggest that the model in this paper can adaptively focus attention on joints highly related to the action to capture crucial motion patterns.

**Figure 14:**
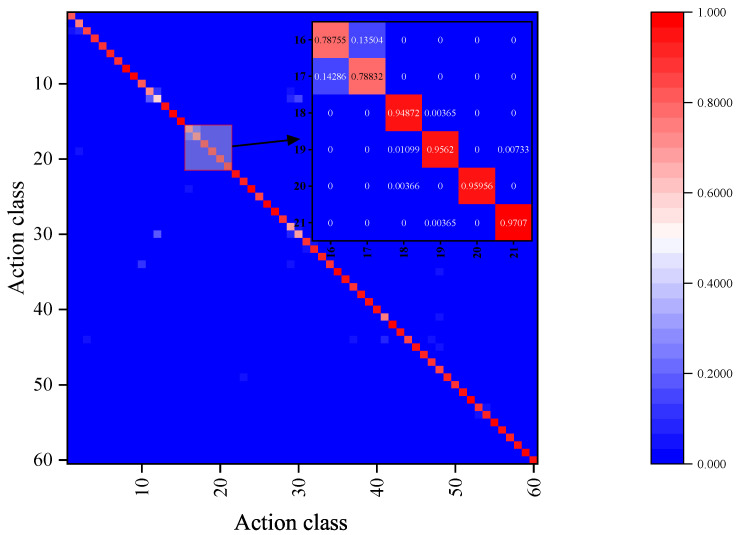
Visualizes the confusion matrix of 60 types of actions in the NTURGBD60 dataset, and a partial zooms in on some of the actions.

As illustrated in Figure 14, the experimental results of the NTU-RGB+D 60 dataset using the xsub protocol were analyzed using a confusion matrix. It is apparent that the proposed model performs well for most actions, with the exception of action A12. writing, having an accuracy below 60%. Conversely, the actions A28. make a phone call/answer phone and A30. typing on a keyboard have an accuracy rate below 70%. However, only subtle differences exist in the hand movements among these three action categories. According to the confusion matrix, not only are similar actions likely to be confused, such as A12. writing and A30. typing on a keyboard, but actions with the same trajectory and those in the opposite direction, such as A16. wear a shoe and A17. take off a shoe, are also susceptible to confusion. This indicates that the model described in this paper is not yet sensitive enough to the temporal sequence of actions.

**Figure 15:**
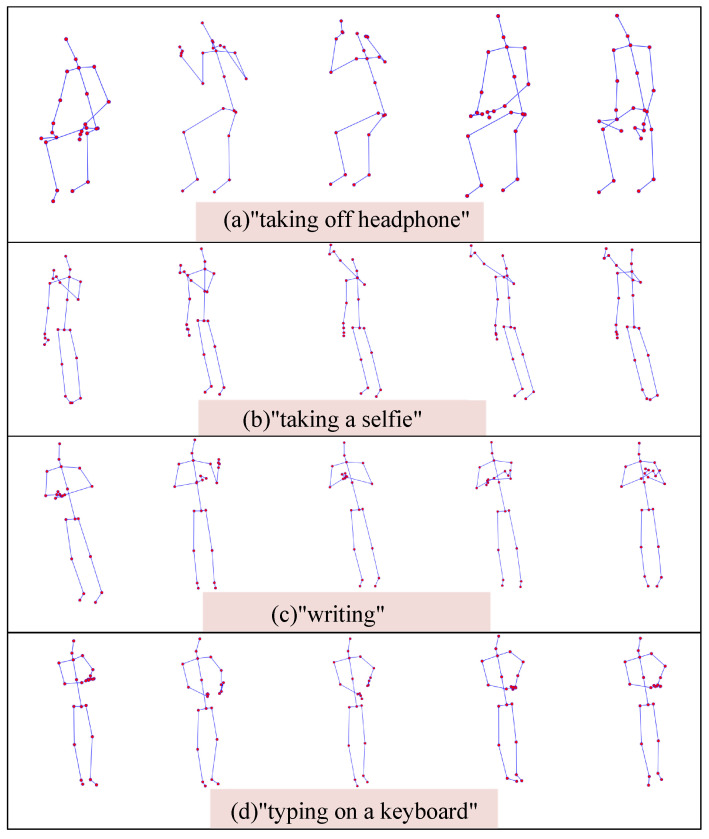
Visualization example of some actions in NTU-RGB+D 60 dataset.

For better understanding, this paper presents several visual examples. Figure 15 illustrates two action examples from Figure 13, as well as a few action examples that could potentially lead to confusion. The subfigures (a) and (b) of Figure 15 correspond to the action examples in Figure 13, reflecting the connecting strength between two action samples. The subfigures (c) and (d) of Figure 15 display two action examples, which, even after being trained with the model proposed in this paper, could still be easily confused. We noticed that these confusing actions visually resemble human movements.

### 4.5. Comparison with the State of the Art

The final model proposed in this paper is compared with existing deep learning methods on two action recognition datasets: NTU-RGB+D 60 and NTU-RGB+D 120. Table 5 below separately presents the recognition accuracy comparison for both datasets. The methods compared include those based on CNN, LSTM, and GCN. Specifically, using the NTURGBD60 dataset, this model was tested on the Xsub and Xview benchmarks, achieving accuracies of 91.9% and 96.3%, respectively. Compared to the methods based on CNNs and LSTM, the GCN model proposed, which utilizes a Multi-level Topological Channel Attention Module and a Multi-scale Global Spatiotemporal Attention Module, significantly outperforms these methods. Compared to the Qin Method [49] and similar techniques, the method proposed in this paper achieves a higher level of accuracy. The accuracy of RSA-Net [50] on the Xsub and Xview benchmarks is 91.8% and 96.8%, respectively. The model proposed in this paper slightly surpasses it on the Xsub benchmark, but cannot exceed the performance of RSA-Net on the Xview benchmark. Compared to other methods that also use attention in the time domain, such as CA-GCN [51] and the Qin Method [52], our model has significant advantages, especially on the Xsub benchmark. For the NTU-RGB+D 120 dataset, our model was tested on the Xsub and Xset benchmarks, achieving accuracies of 88.5% and 90.3%, respectively, surpassing many current methods [50,53,54,55]. For the NW-UCLA dataset, as shown in Table 6 our model achieved superior performance, outperforming most current methods [22,28,56]. The network proposed in this paper performs well in terms of recognition accuracy on the three datasets, benefiting from the utilization of human prior knowledge and motion coordination to weight the skeleton features, as well as the multi-scale feature attention settings for human skeleton positions in the time dimension. This approach effectively encodes the connections between different skeleton nodes, further enhancing the accuracy of action recognition.

## 5. Discussion

In this research, we delve into the significance of our method in skeletal action recognition while analyzing its correlations and differences with existing research, and exploring potential limitations. The primary innovation proposed herein is that the channel and coordination relationships of the human skeleton, along with the temporal positioning features of skeletal nodes, could greatly boost the performance of action recognition. The experiments on challenging datasets like NTU-RGB+D 60, NTU-RGB+D 120, and NW-UCLA have convincingly validated these innovations. Despite the significant success of graph convolution in action recognition as demonstrated by existing research, we find that current models do not fully leverage prior knowledge of human body structure and the coordination between limbs. Therefore, we propose a Multi-level Topological Channel Attention Module based on the human skeleton, integrating limb coordination and skeletal node position features into the model.

In the experimental stage, the model presented in the paper achieved accuracy rates of 91.9% (Xsub) and 96.3% (Xview) on the NTU-RGB+D 60 dataset, surpassing the current mainstream model STF-Net by 0.8% and slightly less than 0.5%, respectively. On the NTU-RGB+D 120 dataset, the model achieved accuracy rates of 88.5% (Xsub) and 90.3% (Xset), outperforming the current mainstream model RSA-Net by 0.1% and 0.6%, respectively. Additionally, the model achieved an accuracy rate of 95.6% on the NW-UCLA dataset, although it did not surpass the current mainstream model CTR-GCN.

This paper acknowledges that notwithstanding the model’s robust performance across all three datasets, it exhibits a notable limitation in detecting actions that follow the same trajectory but in a direction opposite to the original action. Indeed, our model may inaccurately categorize certain complex or unique actions when their trajectories resemble known ones. This constitutes a key limitation of our model, a constraint we recognize and plan to address in future research. We aim to enhance action recognition accuracy by extracting more attributes from human skeletal data.

## 6. Conclusions

In this study, a new action recognition model, leveraging prior human body knowledge and coordination, is proposed. This model takes into account the prior knowledge of human body structure in a granular manner, fully extracting distinctive action features. These features are then processed using Multi-level Topological Channel Attention mechanisms. Moreover, by incorporating the channel relationships of the human skeleton and the coordination of human limbs, the extraction of motion features is enhanced, boosting the discriminative power of the model. The model also includes a Multi-scale Global Spatiotemporal Attention Module, which explores spatiotemporal features of different granularity. It calculates the correlation between the current feature position and other spatiotemporal positions to capture global dependencies. To avoid the issue of non-causality due to future information leakage, the model incorporates a causal convolution block. We verify the efficacy of the model via rigorous testing on three recognized datasets: NTU-RGB+D 60, NTU-RGB+D 120, and NW-UCLA.

## Figures and Tables

**Table 1:** Comparison of the accuracy of different models on the NTU-RGB+D 60 dataset.

MTC	CM	MGS	Xsub (%)	Xview (%)
**✓**	**×**	**✓**	91.6	96.1
**×**	**✓**	**✓**	91.3	95.8
**✓**	**✓**	**×**	91.5	95.7
**✓**	**✓**	**✓**	91.9	96.3

**Table 2:** Comparison of the accuracy of ten types of actions on the NTURGB+D 60 dataset using the CM model.

Action	Xsub			
	Joint (%)	Joint + CM (%)	Bone (%)	Bone + CM (%)
Action3	77.8	78.3 (+0.5)	85.7	86.1 (+0.4)
Action8	95.3	96.0 (+0.7)	95.9	96.7 (+0.8)
Action13	91.1	92.3 (+1.2)	91.7	93.4 (+1.7)
Action22	89.2	91.0 (+1.8)	93.2	96.3 (+3.1)
Action25	81.1	81.3 (+0.2)	82.8	84.3 (+1.5)
Action32	87.4	90.9 (+3.5)	89.9	91.3 (+1.4)
Action34	91.3	91.7 (+0.4)	88.2	88.4 (+0.2)
Action38	93.5	94.6 (+1.1)	93.2	93.5 (+0.3)
Action42	99.5	99.6 (+0.1)	99.4	99.6 (+0.2)
Action52	93.2	95.7 (+2.5)	95.4	96.7 (+1.3)

**Table 3:** Comparison of the accuracy of different methods of constructing coarse-grained ratio maps in the MC module.

Methods	NTU-RGB+D 60	NW-UCLA (%)
	Xsub (%)	
sampling	88.4	92.5
center of gravity	91.5	96.3
average	91.9	96.4

**Table 4:** Comparison of the accuracy of MGS model (including MST and SATM models) on the NTU-RGB+D 60 and NW-UCLA datasets.

MST	SATM	NTU-RGB+D 60 Xsub (%)	NW-UCLA (%)
**×**	**✓**	90.8	96.1
**✓**	**×**	91.7	95.7
**✓**	**✓**	91.9	96.4

**Table 5:** Performance comparison, showing the top-1 accuracy (%) of our proposed method and existing state-of-the-art methods on the NTU-RGB+D 60 and NTU-RGB+D 120 datasets.

Methods	Year	NTU-RGB+D 60		NTU-RGB+D 120	
		Xsub (%)	Xview (%)	Xsub (%)	Xset (%)
DeepLSTM [57]	2017	60.7	67.3	-	-
Temporal ConvNet [58]	2017	74.3	83.1	-	-
Two-stream CNN [18]	2017	83.3	89.3	-	-
ST-GCN [5]	2018	81.5	88.3	-	-
AS-GCN [59]	2019	86.8	94.2	-	-
2s-AGCN [19]	2019	88.5	95.1	82.9	84.9
MS-G3D [32]	2020	91.5	96.2	86.9	88.4
CA-GCN [51]	2020	83.5	91.4	-	-
NAS-GCN [35]	2020	89.4	95.0	-	-
Global-GCN [20]	2021	90.1	95.9	80.9	81.7
ST-GDNs [60]	2021	89.7	95.9	80.8	82.3
Tripool [61]	2021	89.7	95.9	80.1	82.8
SATD-GCN [53]	2022	89.3	95.5	-	-
Qin method [52]	2022	90.5	96.1	85.7	86.8
Qin method [49]	2022	91.6	96.3	88.2	89.2
MSSTNet [62]	2023	89.6	95.3	85.3	86.0
JSCR [54]	2023	88.7	96.1	84.1	86.8
STF-Net [55]	2023	91.1	96.5	86.5	88.2
RSA-Net [50]	2023	91.8	**96.8**	88.4	89.7
Ours (joint)	-	89.3	93.4	84.6	86.2
Ours (bone)	-	90.0	93.5	86.3	87.8
Ours (joint-motion)	-	87.6	92.9	81.3	83.0
Ours (bone-motion)	-	87.3	91.8	81.1	82.9
Ours	-	**91.9**	96.3	**88.5**	**90.3**

**Table 6:** Performance comparison, showing the top-1 accuracy (%) of our proposed method and existing state-of-the-art methods on the NW-UCLA dataset.

Methods	Year	NW-UCLA (Top-1 (%))
Lie Group [63]	2017	74.2
Action ensemble [64]	2017	76.0
Ensemble TS-LSTM [56]	2017	89.2
AGC-LSTM [65]	2020	93.3
Shift-GCN [28]	2021	94.6
CTR-GCN [22]	2022	**96.5**
Ours	-	96.4

## Data Availability

The datasets used in the paper are publicly available and do not require any authorization or permission. Specifically, three datasets are used in the paper: NTU-RGB+D 60 (accessed on 1 May 2023) (https://rose1.ntu.edu.sg/dataset/actionRecognition/), NTU-RGB+D 120 (accessed on 1 May 2023) (https://rose1.ntu.edu.sg/dataset/actionRecognition/) and NW-ULCA (accessed on 1 May 2023) (https://wangjiangb.github.io/my_data.html).
